# Mosaic loss of the Y chromosome in human neurodegenerative and oncological diseases

**DOI:** 10.18699/VJGB-23-61

**Published:** 2023-09

**Authors:** I.L. Kuznetsova, L.I. Uralsky, T.V. Tyazhelova, T.V. Andreeva, E.I. Rogaev

**Affiliations:** Vavilov Institute of General Genetics, Russian Academy of Sciences, Department of Genomics and Human Genetics, Moscow, Russia Sirius University of Science and Technology, Scientific Center for Genetics and Life Sciences, Sochi, Russia; Vavilov Institute of General Genetics, Russian Academy of Sciences, Department of Genomics and Human Genetics, Moscow, Russia Sirius University of Science and Technology, Scientific Center for Genetics and Life Sciences, Sochi, Russia; Vavilov Institute of General Genetics, Russian Academy of Sciences, Department of Genomics and Human Genetics, Moscow, Russia; Vavilov Institute of General Genetics, Russian Academy of Sciences, Department of Genomics and Human Genetics, Moscow, Russia Sirius University of Science and Technology, Scientific Center for Genetics and Life Sciences, Sochi, Russia Lomonosov Moscow State University, Center for Genetics and Genetic Technologies, Faculty of Biology, Moscow, Russia; Vavilov Institute of General Genetics, Russian Academy of Sciences, Department of Genomics and Human Genetics, Moscow, Russia Sirius University of Science and Technology, Scientific Center for Genetics and Life Sciences, Sochi, Russia Lomonosov Moscow State University, Center for Genetics and Genetic Technologies, Faculty of Biology, Moscow, Russia

**Keywords:** mosaic loss of Y chromosome (mLOY), Alzheimer’s disease, GWAS, age-related diseases, oncological diseases, мозаичная потеря Y-хромосомы (mLOY), болезнь Альцгеймера, GWAS, возрастные заболевания, онкологические заболевания

## Abstract

The development of new biomarkers for prediction and early detection of human diseases, as well as for monitoring the response to therapy is one of the most relevant areas of modern human genetics and genomics. Until recently, it was believed that the function of human Y chromosome genes was limited to determining sex and controlling spermatogenesis. Thanks to occurance of large databases of the genome-wide association study (GWAS), there has been a transition to the use of large samples for analyzing genetic changes in both normal and pathological conditions. This has made it possible to assess the association of mosaic aneuploidy of the Y chromosome in somatic cells with a shorter lifespan in men compared to women. Based on data from the UK Biobank, an association was found between mosaic loss of the Y chromosome (mLOY) in peripheral blood leukocytes and the age of men over 70, as well as a number of oncological, cardiac, metabolic, neurodegenerative, and psychiatric diseases. As a result, mLOY in peripheral blood cells has been considered a potential marker of biological age in men and as a marker of certain age-related diseases. Currently, numerous associations have been identified between mLOY and genes based on GWAS and transcriptomes in affected tissues. However, the exact cause of mLOY and the impact and consequences of this phenomenon at the whole organism level have not been established. In particular, it is unclear whether aneuploidy of the Y chromosome in blood cells may affect the development of pathologies that manifest in other organs, such as the brain in Alzheimer’s disease, or whether it is a neutral biomarker of general genomic instability. This review examines the main pathologies and genetic factors associated with mLOY, as well as the hypotheses regarding their interplay. Special attention is given to recent studies on mLOY in brain cells in Alzheimer’s disease.

## Abstract (ru)

Одно из наиболее актуальных направлений современной генетики и геномики человека – разработка новых биомаркеров для прогнозирования и раннего выявления заболеваний человека, а также для мониторинга ответа на терапию. До недавнего времени считалось, что функция генов Y-хромосомы человека ограничена определением пола и контролем сперматогенеза. Благодаря созданию крупных баз данных, полученных на основе метода поиска ассоциаций (GWAS), произошел переход к использованию больших выборок при анализе генетических изменений в норме и при патологиях, в том числе стало возможно оценить связь мозаичной анеуплоидии по Y-хромосоме в соматических клетках с более короткой продолжительностью жизни у мужчин по сравнению с женщинами. На основе данных Британского биобанка (UK Biobank) была обнаружена ассоциация между мозаичной потерей Y-хромосомы (mLOY) в лейкоцитах периферической крови с возрастом мужчин старше 70 лет, а также с рядом онкологических, сердечных, метаболических, нейродегенеративных и психических заболеваний. В результате mLOY в клетках периферической крови стала рассматриваться в качестве потенциального маркера биологического возраста мужчин и как маркер определенных возрастных болезней. На сегодняшний день определено множество ассоциаций между mLOY и генами, выявленными на основании данных GWAS и транскриптомов в пораженных тканях, однако не установлены ни точная причина возникновения mLOY, ни влияние и последствия этого феномена на уровне всего организма. В частности, неясно, влияет ли анеуплоидия по Y-хромосоме в клетках крови на развитие патологий, проявляющихся в других органах, например на мозг при болезни Альцгеймера, или представляет собой нейтральный биомаркер общей геномной нестабильности. В обзоре рассмотрены основные патологии и генетические факторы, ассоциированные с mLOY, и гипотезы их взаимосвязи. Особое внимание уделено последним исследованиям, посвященным mLOY в клетках мозга при болезни Альцгеймера.

## Introduction

Y-chromosome aneuploidy in somatic cells that occurring with age was first identified 60 years ago by karyotyping blood cells (Jacobs et al., 1963) and was attributed to the common age-related phenomenon of accumulation of post-zygotic genetic aberrations of both sex chromosomes and autosomes, unrelated to diseases (Pierre, Hoagland, 1972). Somewhat later karyotyping of bone marrow cells from male patients with acute myeloid leukemia, myelodysplastic syndrome (MDS), myeloproliferative disorders (MPD), and healthy donors was performed to evaluate the contribution of LOY to the malignancy of the diseases (Pierre, Hoagland, 1972, Loss of the Y chromosome…, 1992).

Although aneuploidy is characteristic of many types of tumors, the incidence and level of mLOY both in patients with hematological cancers and healthy donors depend on male age and not on the malignancy of the disease and therefore mLOY cannot be considered a marker of a malignant clone. As a consequence, scientists did not classify mLOY to be a vital factor for several decades, because women survive without the Y chromosome

More recently the hypothesis of the absence of a connection between mLOY and diseases has been challenged in several studies that used the fluorescent in situ hybridization (FISH) method. It was established that the frequency of mLOY is increased in various solid tumors (van Dekken et al., 1990; Sandberg, 1992; König et al., 1994; Takahashi et al., 1994), autoimmune thyroiditis, hematological cancers (Persani et al., 2012), and primary biliary cirrhosis (Lleo et al., 2013). In recent years, with the advent of next-generation sequencing methods and large GWAS databases, including the UK Biobank, with detailed clinical descriptions of donors, it became possible to accurately estimate the frequency of mLOY through statistical analysis. It has been established that 40 % of men over 70 years lack the Y chromosome in more than 5 % of peripheral immune blood cells, and that mLOY in peripheral blood is associated with an increased risk of all-cause mortality (Thompson et al., 2019).

Over the past decade, stable associations were found between mLOY in the blood and various age-related diseases, including hematological (Forsberg et al., 2014; Cáceres et al., 2020) and non-hematological types of cancer (Forsberg et al., 2012, 2014; Cáceres et al., 2020), where the frequency of mLOY varies from 15 to 80 % (Zhang et al., 2007; Bianchi, 2009; Silva Veiga et al., 2012; Duijf et al., 2013), macular degeneration (Forsberg et al., 2014; Cáceres et al., 2020; Duan et al., 2022), cardiovascular diseases (Sano et al., 2022), and neurodegenerative diseases (Dumanski et al., 2016; Vermeulen et al., 2022). In addition, the impact of external factors such as smoking and environmental pollution on mLOY levels in blood cells was revealed (Dumanski et al., 2015; Qin et al., 2019).

Based on these results, it was hypothesized that the biological significance of the Y chromosome goes beyond defining sex and ensuring normal spermatogenesis, and it may contribute to pathological processes, whose molecular mechanisms are yet to be studied. The majority of mLOY studies in pathologies are related to cancer and Alzheimer’s disease, which we will examine in more detail.

## Pathologies associated with mLOY

LOY in solid tumor cells

mLOY has been detected by FISH in various types of stomach cancer, where the frequency of mLOY varied 61–69 % (Hunter et al., 1993; Beuzen et al., 2000), 33–36 % of cases pancreatic cancer (Chia-Hsien Cheng et al., 2001; Kowalski et al., 2007), and 23–34 % of cases of bladder cancer (Sauter et al., 1995; Minner et al., 2010), and in 12.7 % of breast cancer in men (Agahozo et al., 2020). LOY in various types of renal cell carcinoma has been well studied using FISH, where the frequency of LOY varies from 77 % in papillary renal cell carcinoma to 39 % in the most common type – clear cell renal cell carcinoma (Büscheck et al., 2021).

It has been suggested that aberrations of the Y chromosome may be responsible for the higher incidence of clear cell renal cell carcinoma in men compared to women. Indeed, in addition to the previously known association of copy number variations in autosomes and this disease, analysis of whole-genome data and multiplex PCR have shown that LOY is detected in tumor cells of more than 30 % of male patients (Arseneault et al., 2017). Surprisingly, the frequency of LOY in prostate cancer tumor samples was found to be very low. For example, in a study involving of 2053 specimens from patients who underwent prostatectomy, only 12 cases of Y chromosome aneuploidy were detected (Stahl et al., 2012). This low frequency of LOY may be explained by the fact that mLOY reflects the overall chromosomal instability of tumor cells, which is lower in prostate cancer compared to many other types of cancer (de Matos et al., 2019).

Due to the prevalence of LOY in blood, it was expected to detect an increased frequency of LOY in bone marrow samples from patients with various hematological disorders. In the study of 237 patient samples, including those with MDS, MPN, acute myeloid leukemia, chronic myeloid leukemia, multiple myeloma, and lymphoma, LOY was detected in only 10 % of cases (Zhang et al., 2007).

Unfortunately, the prognostic potential of LOY in tumors is not entirely clear. In head and neck cancer, LOY in tumor cells may be an indicator of therapy resistance (Hollows et al., 2019). In invasive urothelial carcinomas, LOY in tumors was not associated with survival, increased risk of recurrence, or increased risk of progression (Minner et al., 2010). For example, in prostate cancer, no significant associations were found between loss of the Y chromosome in tumor cells and patient age, tumor stage, or risk of recurrence. However, it was significantly associated with a high Gleason score, which is associated with a poor prognosis (Stahl et al., 2012).

In summary, the absence of the Y chromosome is a common event in tumors, but it significantly differs in frequency for different types of cancer, and not enough data have yet been accumulated to understand the reasons for these differences. In other words, the presence and level of mLOY in tumors cannot yet be considered as a marker of malignancy or important information for predicting treatment and recurrence.

## mLOY in peripheral blood in hematological and non-hematological cancers

A study of a large cohort from the Uppsala Longitudinal Study of Adult Men (ULSAM) database showed that men with mLOY in blood had an increased risk of cancer diagnosis and mortality from various non-hematological types of cancer (Forsberg et al., 2014). An analysis including data for all solid tumor cases submitted to the UK Biobank was also conducted, with results confirming an association between mLOY frequency and tumor presence (Loftfield et al., 2019). The association of mLOY in blood with the risk of cancer in other organs has been described in several independent studies on a smaller cohort, for example, an increased frequency of mLOY compared to age norms was shown for prostate cancer and colorectal cancer (Noveski et al., 2016).

Loss of the Y chromosome in leukocytes can also act as a marker of a tumor clone in hematological diseases. For example, associations of mLOY with a worse prognosis in leukemias such as AML (Holmes et al., 1985), chronic myeloid leukemia (Lippert et al., 2010), and chronic lymphocytic leukemia (Chapiro et al., 2014). However, in the case of MDS that has not progressed to AML, the presence of mLOY may be a favorable factor for recovery (García-Isidoro et al., 1997). Another identified condition for a stable association with MDS and the ability to predict its course is the presence of mLOY in a very high proportion (more than 75 %) of blood cells (Wiktor et al., 2000; Ouseph et al., 2021).

An attempt to narrow the search area to specific types of cells in the case of MDS was undertaken in the work (Ganster et al., 2015), where the ratio of the level of mLOY in CD34+ cells associated with MDS and mLOY in CD3+ cells not associated with MDS was analyzed. The results showed that the level of mLOY in both CD34+ and CD3+ cells were agedependent in men without hematological diseases, and in the case of CD34+ cells, it was significantly higher in MDS patients compared to elderly control men without hematological pathologies. These data indicate that the level of LOY in the blood has an age-related basis, but is also associated with MDS. In addition, the authors determined the threshold level of LOY in CD34+ cells in peripheral blood to distinguish age-related changes from increased mLOY in MDS, which was 21.5 %.

Interesting data on a Chinese cohort were obtained in a study of mLOY in lung cancer, where it is a protective factor against the development of lung cancer, but only in non-smoking patients (Qin et al., 2019), which is the only report on a possible protective factor of mLOY, and is difficult to interpret.

Additionally, it is worth noting the results of data analysis from the UK Biobank, contradicting the above, which concluded that mLOY is primarily associated with age and smoking, but not with common types of cancer (Zhou et al., 2016). This conclusion was criticized, as the study used data not only from leukocytes but also from buccal epithelial cells (Forsberg et al., 2019). Later, it was shown that the addition of buccal epithelial cell data to mLOY analysis negated the result obtained for mLOY in leukocytes (Zhou et al., 2016). Thus, despite some disagreements in the results of the studies, it could be concluded that mLOY in human blood has significant potential as one of the biomarkers of malignant diseases, especially when the level of mosaicism is above 20 %.

## Mechanism of mLOY geneses

The results of cytological studies suggest that the loss of the Y chromosome in somatic cells occurs during cell division. The most common theory is that mLOY is associated with a mechanism involving the formation of micronuclei with isolated Y chromosomes after improper chromosome segregation during mitosis, followed by the destruction of the micronucleus through autophagy, leading to the appearance of 45,X0 cells in older men (Guttenbach et al., 1994; Ly et al., 2019; Guo et al., 2020). Age-related factors, such as telomere shortening and centromere dysfunction, may increase chromosomal segregation errors. Compared to mosaic aneuploidy of autosomes and the X chromosome, mLOY is a more frequent event, presumably due to its overall high heterochromatinized status and small size (Clark, 2014; Wright et al., 2017). In addition, the human Y chromosome is enriched in repetitive sequences, which may play a role in chromosome segregation errors during mitosis (Jobling, Tyler-Smith, 2017).

It remains unclear at what stage of human leukocyte differentiation the loss of the Y chromosome occurs and the clone having a 45,X0 karyotype is formed. One hypothesis is related to primary loss of the Y chromosome in hematopoietic precursors. This process can be associated with clonal hematopoiesis of indeterminate potential (CHIP), which is defined as the detection of somatic mutations in genes typically associated with myeloid neoplasms in individuals without signs of hematologic malignancy. CHIP occurs due to aging of hematopoietic stem cells that have accumulated mutations, leading to proliferative advantage over their peers, and as a result, to clonal expansion (Genovese et al., 2014; Jaiswal et al., 2014).

CHIP is an age-related phenomenon, regularly observed in healthy elderly individuals with a frequency of up to 10 % at the age of 70, and CHIP is also associated with an increased risk of hematologic malignancies and cardiovascular disease (Genovese et al., 2014; Jaiswal et al., 2014, 2017). Whether mLOY is one of the manifestations associated with CHIP has been considered in the work of V. Ljungström et al. (2022), where the frequency of mutations and mLOY in monocytes of men aged 65–90 was analyzed. The results of the study indicate a frequent coexistence of mLOY and CHIP in monocytes. Another study also revealed the co-occurrence of LOY and CHIP in bone marrow cells obtained from patients referred for clinical bone marrow examination (Ouseph et al., 2021). It should be noted that in the case of the monocyte study, cases of LOY without CHIP, and vice versa, were also detected. However, the sample size of both studies was very limited (24 and 73 participants), and the obtained result requires confirmation in further studies. It should also be taken into account that CHIP is observed in 10 % of 70-year-old men, and mLOY – in 40 %.

We should also note that mLOY stands out among aneuploidies in leukocytes by a higher frequency of occurrence in compared to other chromosomes, and may apparently be a biomarker of overall chromosomal instability, which is characteristic of general aging of the organism and many human diseases. However, the use of mLOY for diagnostic and prognostic purposes is premature due to the lack of precise mechanistic data on the nature of this phenomenon. It is also yet to be determined whether the observed level of mLOY depends on de novo events or high clonality, and how both possible mechanisms are related to stages of different diseases.

## Genetic factors of mLOY

The human Y chromosome, according to the latest version (T2T-CHM13v2.0, hereafter T2T-Y) of the human genome assembly is over 62 million base pairs long (Rhie et al., 2022). The Y chromosome consists of three main regions: the ends of the chromosome contain pseudoautosomal regions (PAR1 and PAR2) that are homologous to the ends of the X chromosome, and the remainder (about 95 %) is the male-specific region of the Y chromosome (MSY), which does not undergo recombination (Colaco, Modi, 2018), resulting in the accumulation of repetitive sequences in the MSY.

According to the T2T-Y assembly, the Y chromosome contains 693 annotated genes and 888 transcripts, of which 107 (493 transcripts) are protein-coding. In addition to all the genes annotated in GRCh38-Y, the T2T-Y assembly contains 110 genes, of which 42 are predicted to be protein-coding. Y genes constantly degrade, which may be due to the lack of recombination on this chromosome. The presence of a large number of repeats, in turn, contributes to chromosomal rearrangements and intrachromosomal recombination (Jobling, Tyler-Smith, 2003; de Knijff, 2022). Unfortunately, with the advent of the GWAS era, Y chromosome variants have not been included in most GWAS due to the lack of recombination and high repeat content (Xue, Tyler-Smith, 2017; Parker et al., 2020), making the Y chromosome much less characterized in molecular genetic terms than other human chromosomes.

To identify the molecular genetic factors underlying the occurrence of mLOY in a certain proportion of men, GWAS data for autosomes are primarily used. The first genetic association discovered with mLOY was linked to a single nucleotide variation rs2887399 near the TCL1A gene (Zhou et al., 2016). The product of this gene, the TCL1A protein, is involved in carcinogenesis, mainly through chromosomal rearrangements (Laine et al., 2000). A strong association between rs2887399 and mLOY has been replicated in subsequent studies on other cohorts (Wright et al., 2017; Thompson et al., 2019). In total, over 150 genetic variants in autosomes associated with mLOY have been found (Thompson et al., 2019), including variants in genes involved in the regulation of cell cycle (CCND2, CCND3, CDKN1B, CDKN1C, CDK5RAP1, ATM), chromatin structure during mitosis (NCAPG2, SMC2), and kinetochore structure and function (CENPN, CENPU, PMF1, ZWILCH, SPDL1).

Associations with cancer susceptibility genes, as well as somatic tumor growth factors and anti-tumor therapy targets, have also been identified. Such genes include those encoding proteins involved in DNA damage response (SETD2, DDB2, PARP1, ATM, TP53, and CHEK2) and in apoptotic processes (PMAIP1, SPOP, LTBR, SGMS1, TP53INP1, DAP, BCL-2 family genes). These associations were confirmed in Japanese and European populations, collectively containing data for over 750,000 men (Thompson et al., 2019), and these variants have later been successfully used to predict mLOY using polygenic risk score estimates (Riaz et al., 2021).

The identified genetic variations in key cell cycle genes provide evidence that cells without a Y chromosome may avoid molecular processes that destroy aneuploid cells, resulting in their proliferation and accumulation in the tissues. Moreover, there are no genes on the Y chromosome associated with somatic cell survival or mitosis, so its absence should not be associated with limitation on cell division (Maan et al., 2017). On the other hand, many of the most commonly observed mutations associated with LOY are linked to general genomic instability. Indeed, based on data from a large-scale cohort GWAS, it has been shown that mosaicisms across autosomes are more common in men with LOY (Zhou et al., 2016).

Most of the mLOY-related genetic variants are often located near genes known as tumor growth encoding factors, targets for cancer treatment, or those that contribute to cancer susceptibility. This is one of the explanations for the association between mLOY and non-hematological cancers and the identified loci are associated not only with various types of male-specific cancers (prostate cancer and germ cell tumors), but also with gender-independent types of cancer (lung cancer, colorectal cancer, glioma, and renal cell carcinoma), as well as with an increased risk of developing specific nonhematological types of cancer in women (breast, ovarian, and endometrial cancer) (Thompson et al., 2019). Based on these results, it can be concluded that mLOY reflects a certain common autosomally determined state of the organism.

In addition to elucidating the root cause of mLOY, the challenge of genetic research on this phenomenon is to identify the consequences of mosaic absence of all genes on the sex chromosome in leukocytes in an elderly man. One hypothesis is that the loss of certain genes in connection with clonal absence of the Y chromosome can be considered as a trigger of molecular and biological processes that lead to age-related pathologies. In particular, it can be assumed that the loss of genes in the PAR1 and PAR2 regions may affect the level of expression of these genes.

One example is the CD99 gene, located in the PAR1 region of the Y and X chromosomes, which is not subject to X-inactivation in women (Sharp et al., 2011), which may indicate the importance of its balanced expression. The CD99 gene, most highly expressed in blood cells, encodes the transmembrane glycoprotein CD99, playing an important role in the functioning of the immune system and affects the key properties of leukocytes (Sohn et al., 2001; Schenkel et al., 2002; Brémond et al., 2009; Pasello et al., 2018). Indeed, CITESeq analysis uncovered a reduced surface expression of CD99 in individual leukocytes lacking a Y chromosome, which may be indicative of a link between LOY and immune functions of leukocytes that is dependent on the homologous regions of sex chromosomes (Mattisson et al., 2021).

Another Y chromosome gene that can directly affect the development of malignant tumors is KDM6C, located in the MSY region and encoding histone demethylase. KDM6C has a functionally similar X-linked homolog, KDM6A, whose deficiency is particularly associated with the progression of clear cell renal cell carcinoma (Arseneault et al., 2017). Other interesting Y chromosome genes functionally corresponding to tumor suppressors are KDM5B and KDM5D (histone demethylases), DDX3Y (RNA helicase), EIF1AY (translation initiation factor), RPS4Y1 (ribosome subunit), ZFY (transcription factor) (Dunford et al., 2017; Willis-Owen et al., 2021).

The development of omics technologies has also made it possible to consider in more detail the potential additional functions of Y chromosome genes. Thus, based on proteomic studies, it was found that the DDX3Y gene, located in the MSY region of the Y chromosome, can modulate neuronal differentiation (Vakilian et al., 2015), and the Y chromosome haplogroup may be a risk factor for prostate cancer (Cannon- Albright et al., 2014). It has been discovered that SRY (the MSY region that determines sex) may be an oncogenic factor (Murakami et al., 2014), and furthermore, SRY is involved in the molecular genetic pathway associated with pulmonary arterial hypertension (Yan et al., 2018).

There are initial indications of a possible effect of mLOY on autosomal gene expression. In particular, increased expression of the known oncogene TCL1A was detected based on single cell transcript sequencing (scRNAseq) data. The product of this gene, the TCL1 protein, is a stimulator of cell proliferation (Thompson et al., 2019). Tumors with mLOY show increased expression of genes involved in resistance to both radiation therapy and platinum-based chemotherapy drugs (Hollows et al., 2019), which partly explains the association of mLOY with treatment prognosis.

Thus, genetic studies suggest that mLOY is determined as a highly polygenic phenomenon. Unfortunately, it is difficult to trace from GWAS data whether single-nucleotide and structural variations in the Y chromosome itself influence the risk of mLOY. In particular, it will be relevant for population studies to determine the relationship between Y chromosome haplogroups and various mLOY indicators. Based on the genetic factors identified through association analysis, it can be concluded that mLOY is influenced by an increase in the frequency of errors during mitosis and a disruption of chromosomal balance recognition and apoptotic regulation.

## mLOY in Alzheimer’s disease

Alzheimer’s disease is a progressive and irreversible neurodegenerative disorder of the central nervous system, responsible for approximately 70 % of all dementia cases. Mutations in the PSEN1, PSEN2, and APP genes were found as the main cause of familial AD (Sorbi et al., 1995; Masters et al., 2015).

Many variations in genes that are risk factors for sporadic AD are also known. Among these, the apolipopro- tein E4 (ApoE4) allele is the greatest genetic risk factor, with homozygous ApoE4 carriers having a 14-fold increased susceptibility to AD (Yamazaki et al., 2019). Although the average lifespan of men is shorter than that of women, this age-related fatal disease develops less frequently in men. Therefore, the influence of the male sex chromosome on AD pathogenesis was unexpected. However, the results of the first case-control study of mLOY in AD patients using the European Alzheimer’s Disease Initiative stage 1, ULSAM, and the Prospective Study of the Vasculature in Uppsala Seniors (PIVUS) database showed that mLOY is statistically significantly 2.8 times more common in leukocytes of men with AD compared to a control group without brain pathologies (Dumanski et al., 2016). These results were based on the analysis of high-quality sequencing data from 606 blood DNA samples from AD patients and 1005 control samples of all ages. To minimize the influence of age component on both AD and mLOY (risk), separate confirmation of the association between AD and mLOY was obtained for two sub-samples with narrow age ranges: men aged 70–75 and 75–80 years (Dumanski et al., 2016). The identified association persisted even after taking into account the influence of other agerelated diseases (cardiovascular disease, diabetes), as well as unhealthy habits (alcohol and smoking). Longitudinal studies, in which participants were tested several times at an interval of about 10 years, allowed the evaluation of mLOY as a risk factor for AD. Thus, during the observation period of ULSAM and PIVUS, 140 individual cases of AD were registered. The results of the data analysis for these individuals, adjusted for the age of the patient at the moment of blood sampling and age-related diseases, showed that mLOY is a significant risk factor for AD, increasing the likelihood of its diagnosis by 6.8-fold

Since the ApoE4 haplotype is one of the most important risk factors for the development of AD and currently the only confirmed genetic factor that affects lifespan on multiple independent samples (Nebel et al., 2011), its contribution is increasingly being evaluated in studies related to age-related diseases. It is known that the presence of the ApoE2/3/4 haplotype can affect the phenotypic expression of other genetic variants (Kuznetsova et al., 2022). In the case of mLOY, it has been shown that, firstly, the ApoE2/3/4 haplotype does not affect the level of mLOY in leukocytes (Dumanski et al., 2016). Also, the assessment of the joint effect of ApoE4 and mLOY gave a negative result, from which the authors concluded that the risk of developing AD from these factors manifests independently

However, some studies suggest that an integrative effect of mLOY and genes related to the development of AD is possible. Thus, the influence of the ApoE4 haplotype on the presence of mLOY in dorsolateral prefrontal cortex cells was revealed (Graham et al., 2019), and in a study using neurons obtained from induced pluripotent stem cells from a patient with familial AD with a mutation in the PSEN1 gene, it was demonstrated that LOY enhances the toxic effects of Aβ42, leading to impared neuron differentiation and premature cell death (Mendivil-Perez et al., 2019). In addition, a statistically significant correlation between mLOY and variation located in proximity to the SPON1 gene, which is associated with the severity of dementia, was demonstrated in the Japanese population (Sherva et al., 2014; Terao et al., 2019).

An attempt to answer the key question of whether LOY is a cause or consequence of age-related diseases has also been undertaken for Alzheimer’s disease. Quantitative comparisons of LOY frequency showed that the percentage of mLOY is higher in leukocytes than in neurons (Graham et al., 2019), and is also associated with age. Studies using cell lines have shown that the absence of the Y chromosome is more commonly observed in fibroblasts than in neuron-like iPS cells. However, considering that LOY may depend on the proliferative capacity of cells, microglia and oligodendrocyte precursor cells may be more prone to LOY accumulation than terminally differentiated neuronal cells.

Based on scRNAseq data and single-nucleus RNA sequencing by (Vermeulen et al., 2022), LOY enrichment was shown in microglia, as opposed to neurons, astrocytes, and oligodendrocytes, in non-demented patients significantly higher mLOY levels in observed microglia in case of Alzheimer’s disease. Comparative analysis of microglia in different brain regions revealed that in the somatosensory cortex of male AD patients 21 % of microglia classified as LOY compared to 1.81 % in controls, while in the entorhinal cortex, the frequency of LOY was 32.7 % in patients and 3.27 % in controls. According to the authors, the elevated level of LOY in the entorhinal cortex of AD patients is of particular interest because this part of the brain is the first to be affected in AD (Gómez-Isla et al., 1996; Kobro-Flatmoen et al., 2021).

Thus, based on several studies, it is becoming increasingly evident that the frequency of mLOY is elevated in AD and cancer, and is also a risk factor for these conditions (Forsberg et al., 2014; Dumanski et al., 2016; Noveski et al., 2016). A dual pathway of development for certain triggers leading to either cancer or AD has been noted in several works (Behrens et al., 2009; Lanni et al., 2021). Indeed, it has been shown that mLOY in blood is a competing risk factor between the onset of AD and solid cancers (Dumanski et al., 2016).

Summing up these results, it could be concluded that mLOY in blood, being a male-specific risk factor for both AD and cancer, may at least partially explain why men on average live shorter lives than women.

Recent studies have provided the first insights into changes in gene expression in brain cells associated with mLOY (Graham et al., 2019). Analysis using a population sample of differentially expressed genes in microglial cells with and without the Y chromosome revealed 193 genes with dysregulated expression upon Y chromosome loss, including genes involved in aging, basic glioma biology, and inflammation (Vermulen et al., 2022). Through the intersection of the list of autosomal genes with dysregulated expression influenced by mLOY in leukocytes and microglia, genes TMEM176B, S100Z, TMEM71, CD226, B2M, SCMH1, LITAF, and IL15, predominantly related to immune response and inflammation, were identified.

For genes located in the PARs of the sex chromosomes, a correlation was found between mLOY and the expression of CD99, previously identified in leukocytes, supporting the hypothesis of a possible disruption in immune system functioning directly associated with Y chromosome loss. In addition to the CD99 gene, dysregulation of the genes GTPBP6, IL3RA, SLC25A6, P2RY8, AKAP17A, DHRSX, and CSF2RA, located in the PAR1 region of the Y chromosome, was observed in microglia. In particular, the CSF2RA gene, which is involved in a molecular pathway associated with neurodegeneration, is expressed only in microglia and macrophages associated with the brain. As this study was conducted by a single research group, these findings require confirmation in independent studies.

The association of mLOY in brain cells with AD prompted investigations into the link between this phenomenon and psychiatric disorders. Data have been obtained indicating either no association or weak association with schizophrenia (Hirata et al., 2018), as well as strong association of mLOY with suicidal tendencies, with blood mLOY levels being almost three times higher in the latter case. Interestingly, no changes in mLOY levels were detected in the dorsolateral prefrontal cortex of postmortem brains of individuals who died by suicide, but these data are limited in reliability due to the small sample size (Kimura et al., 2018).

## Conclusion

Numerous studies in large cohorts have shown that the LOY in blood cells is a significant risk factor for mortality and various diseases in men. From the accumulated data on associations of mLOY with various pathologies, it becomes increasingly likely that analysis of LOY may become a sensitive biomarker for AD, solid tumors, hematological malignancies, and overall genomic instability. However, it should be kept in mind that no study has provided direct evidence on how mLOY arises, how it affects cells, and what consequences it has. In terms of a predictive potential perspective, it is necessary to understand whether mLOY is a barometer reflecting the presence of pathologies or, conversely, whether mLOY arises de novo and participates in the pathogenesis. In this regard, there are several questions that need to be answered, such as whether the pathogenic effect of LOY manifests in untransformed blood cells or in transformed clones in the case of hematological malignancies.

Another question that needs to be resolved is how LOY in normal blood cells can be linked to pathological processes in other organs, leading to tumors in other organs or neurodegeneration in the brain. Currently, the most attractive hypothesis is the immune surveillance hypothesis, explaining the mechanisms underlying associations between LOY in blood cells and enhanced neoplastic cell proliferation in tumors in other parts of the body or the development of Alzheimer’s disease.

Comparisons of data for mLOY in different populations are limited by a small number of studies to date and differences in methods. Most of the obtained data are ethnically limited and may not be confirmed in poorly represented populations in databases, such as African or Middle Eastern populations. Based on two studies from the same research group, it can be preliminarily concluded that the prevalence of mLOY in men is higher in European populations than in African populations (Loftfield et al., 2018, 2019). However, it should be noted that such comparisons are currently limited.

Despite the many questions that are likely to be actively addressed in connection with the popularity of this topic, it can already be concluded that mLOY plays a role in determining the health of elderly men.

## Conflict of interest

The authors declare no conflict of interest.
